# Feasibility and Outcomes of Percutaneous Coronary Intervention After TAVI: A Comparison Between BEV and SEV Platforms

**DOI:** 10.3390/jcm15145616

**Published:** 2026-07-17

**Authors:** Ziad Arow, Omar Oliva, Juri Iwata, Akiko Masumoto, Arthur Clement, Vittorio Zuccarelli, Antonella Millin, Abid Assali, Nicolas Dumonteil, Didier Tchetche, Chiara De Biase

**Affiliations:** 1 Groupe CardioVasculaire Interventionnel, Clinique Pasteur, 31076 Toulouse, France; ziad.arow@gmail.com (Z.A.); omaroliva93@gmail.com (O.O.); juriandbbpomecr@gmail.com (J.I.); akikomasu@gmail.com (A.M.); arthur.clementvt@gmail.com (A.C.); zuccarellivittorio@gmail.com (V.Z.); antonella.millin@gmail.com (A.M.); ndumonteil@clinique-pasteur.com (N.D.); dtchetche@clinique-pasteur.com (D.T.); 2 Cardiology Department, Meir Medical Center, Tel Aviv University, Tel Aviv-Yafo 6997801, Israel; aassali@clalit.org.il; 3 Interventional Cardiology Unit, IRCCS Multimedica, 20099 Sesto San Giovanni, Italy

**Keywords:** transcatheter aortic valve implantation (TAVI), balloon-expandable valve (BEV), self-expandable valve (SEV), chronic coronary syndrome (CCS), acute coronary syndrome (ACS), percutaneous coronary intervention (PCI)

## Abstract

**Background**: Transcatheter aortic valve implantation (TAVI) has become an established therapy for severe aortic stenosis and is increasingly performed in younger and lower-risk patients. Coronary access following TAVI may be technically challenging, particularly with long stent supra-annular self-expandable platforms. We aimed to evaluate the procedural characteristics and clinical outcomes of percutaneous coronary intervention (PCI) after TAVI, comparing patients treated with balloon-expandable valves (BEVs) and self-expandable valves (SEVs) at our center. **Methods**: In this retrospective single-center study, we included consecutive patients who underwent TAVI with BEVs (Edwards Sapien 3 and sapien 3 Ultra platforms) or SEVs (Medtronic Evolut R, Evolut Pro, Evolut Pro+, Evolut Fx and Abbot Navitor platforms) between 2016 and 2024 and subsequently underwent PCI in the index procedure post valve implantation, or returned for PCI due to chronic coronary syndrome (CCS) or acute coronary syndrome (ACS). Simple diagnostic coronary angiography without PCI was not considered in this analysis. The primary outcome was PCI success and procedural characteristics according to valve platform. A prespecified subgroup analysis was performed according to clinical presentation (CCS vs. ACS). Clinical outcomes were analysed in the whole population. **Results**: A total of 73 patients underwent PCI after TAVI, including 34 with balloon-expandable (Sapien) and 39 with self-expandable (33 Evolut and 6 Navitor) valves. The mean age was 81 ± 6 years, and 64% were male. The median time from TAVI to PCI was 470 days. LAD PCI was numerically more frequent in the BEV group (59% vs. 41%, *p* = 0.129), whereas LCX PCI was more common in the SEV group (54% vs. 29%, *p* = 0.035). PCI success was high overall (95%) and did not differ significantly between BEV and SEV platforms (97% vs. 92%, *p* = 0.374). Final TIMI 3 flow was achieved in 99% of cases. Intraprocedural coronary complications were infrequent (5%) and did not differ significantly between platforms. At 1 year, overall mortality was 8%, with no significant difference between BEV and SEV platforms. In a subgroup analysis, PCI success was high in CCS and ACS presentations (97% vs. 91%, *p* = 0.218), with a trend of higher 1-year mortality in ACS compared with CCS (15% vs. 3%, *p* = 0.061). **Conclusions**: PCI after TAVI is highly feasible, with excellent angiographic success and low complication rates. Procedural and clinical outcomes were comparable between BEV and SEV platforms.

## 1. Introduction

Transcatheter aortic valve implantation (TAVI) has transformed the management of severe aortic stenosis, evolving into a life-saving therapeutic option [1,2,3,4]. In the past decade, its global use has increased substantially, supported by expanding indications and continuous improvements in device design and procedural techniques [5,6]. As TAVI is increasingly performed in younger and lower-risk patients, the long-term management of concomitant coronary artery disease (CAD) has become an important clinical consideration [7,8,9]. A substantial proportion of patients undergoing TAVI have pre-existing CAD, and many will require coronary angiography or percutaneous coronary intervention (PCI) during follow-up [10,11,12]. However, coronary access after TAVI may be technically challenging, particularly with supra-annular self-expandable platforms due to their frame architecture and leaflet position [13,14,15], and data addressing this issue remain limited in the literature.

As the TAVI population continues to grow, understanding the safety, procedural complexity, and outcomes of PCI performed after TAVI has greater clinical relevance. The aim of this study was to evaluate the procedural challenges and clinical outcomes of PCI performed after TAVI in a real-world cohort, comparing balloon-expandable valves (BEVs, Sapien platforms) and self-expandable valves (SEVs, Evolut and Navitor platforms).

## 2. Methods

This retrospective, single-center study was conducted at Clinique Pasteur in Toulouse, France. We included consecutive patients who underwent TAVI for severe aortic stenosis with either BEVs (Sapien 3 and Sapien 3 Ultra, Edwards Lifesciences, Irvine, CA, USA) or SEVs (Evolut R, Evolut Pro, Evolut Pro+, Evolut Fx, Medtronic, Minneapolis, MN, USA; and Navitor, Abbott, Abbott Park, IL, USA) between 2016 and 2024 and subsequently underwent PCI either during the index TAVI procedure or later during follow-up. Because only six patients received a Navitor valve, all self-expandable platforms were analyzed as a single SEV group. In cases of concomitant PCI during the index procedure, coronary intervention was performed after valve implantation in all patients. All PCI procedures were performed by experienced interventional cardiologists at a high-volume TAVI center with expertise in coronary intervention following transcatheter valve implantation. Acute coronary syndrome (ACS) was defined as unstable angina (UA), non-ST-segment elevation myocardial infarction (NSTEMI), or ST-segment elevation myocardial infarction (STEMI). Patients undergoing diagnostic coronary angiography without PCI were excluded, since coronary cannulation only was not part of this analysis. The primary objective of the study was to evaluate the procedural characteristics and clinical outcomes of PCI after TAVI rather than coronary angiography alone. The analysis was performed at the patient level. Patients undergoing multivessel PCI during the same procedure were included once, and procedural characteristics and clinical outcomes were analyzed per patient. Patient selection for TAVI followed the European Society of Cardiology guidelines for the management of valvular heart disease and was determined by a multidisciplinary Heart Team [1]. The study was approved by the local ethics committee. Given the retrospective nature of the study and the use of anonymized registry data, the requirement for informed consent was waived in accordance with national regulations.

The primary endpoint of the study was procedural PCI success after TAVI. Secondary endpoints included procedural characteristics, intraprocedural complications, in-hospital outcomes, and 1-year all-cause mortality in the overall cohort and according to valve platform. PCI success after TAVI was defined as successful stent implantation or balloon angioplasty with drug-coated balloon with final TIMI 3 flow and no intraprocedural coronary complications. Intraprocedural coronary complications were defined as a composite of in-stent thrombosis, slow or no-reflow, distal embolization, coronary dissection, coronary perforation, or intra-procedural death. Although intraprocedural coronary complications were incorporated into the composite definition of PCI success, they were also reported separately to describe the type and frequency of individual procedural events. Major bleeding was defined according to the Valve Academic Research Consortium-2 (VARC-2) criteria. Follow-up for all-cause mortality at 1 year was complete for the entire study cohort. In addition, a prespecified subgroup analysis was performed to assess procedural and clinical outcomes according to clinical presentation (CCS vs. ACS).

### Statistical Analysis

Categorical and binary variables are presented as frequencies and percentages, with comparisons between groups performed using the Pearson chi-square test or Fisher’s exact test, as appropriate. Continuous variables were assessed for distribution. Normally distributed variables are reported as mean ± standard deviation and were compared using the unpaired two-tailed Student’s *t*-test. Variables not following a normal distribution are expressed as median with interquartile range and were analyzed using the Mann–Whitney U test. The Kruskal–Wallis test was used to evaluate differences in the distribution of continuous variables.

A subgroup analysis was conducted to assess procedural and clinical outcomes according to clinical presentation (CCS vs. ACS). All statistical tests were two-tailed, and a *p*-value < 0.05 was considered statistically significant. Statistical analyses were performed using SPSS software, version 29.0.2.0 (IBM Corp., Armonk, NY, USA).

## 3. Results

### 3.1. Baseline Characteristics

A total of 73 patients who underwent PCI after TAVI were included in the study, of whom 34 had received a balloon-expandable valve (Sapien) and 39 a self-expandable valve (33 Evolut and 6 Navitor). Baseline characteristics are summarized in Table 1. The overall mean age was 81 ± 6 years, and 64% of patients were male. Patients in the SEV group exhibited numerically higher rates of dyslipidaemia (51% vs. 44%), diabetes mellitus (30% vs. 21%), coronary artery disease (78% vs. 68%), prior PCI (54% vs. 32%), and prior CABG (30% vs. 15%), although none of these differences reached statistical significance. In contrast, atrial fibrillation was numerically more frequent in the BEV group (23% vs. 15%, *p* = 0.378). Surgical risk scores tended to be higher in the SEV group, with a median EuroSCORE II of 5.2 vs. 5.0 (*p* = 0.167) and a median STS score of 4.8 vs. 3.8 (*p* = 0.151).

TAVI procedural characteristics are summarized in Table 2. The vast majority of patients underwent TAVI via transfemoral access (97%), with similar rates between groups. Device success was high and comparable in both groups (91% vs. 92%, *p* = 0.861). Valve size distribution differed between platforms. The BEV group more frequently received 26-mm valves (59% vs. 21%, *p* = 0.001), whereas the SEV group more commonly received 29-mm valves (46% vs. 26%, *p* = 0.082). Concomitant PCI during TAVI was performed in 4 (5%) of all patients.

The overall post-TAVI mean transvalvular gradient was 10 ± 4 mmHg and was significantly higher in the BEV group compared with the SEV group (12 ± 4 vs. 8 ± 4 mmHg, *p* < 0.001). Left ventricular ejection fraction after TAVI was similar between groups. Periprocedural stroke was infrequent (3% overall), and rates of permanent pacemaker implantation did not differ significantly between groups.

### 3.2. PCI Procedural Characteristics

PCI procedural characteristics are summarized in Table 3. PCI was performed for CCS in 55% of patients and for ACS in 45%, with no significant difference between valve groups. The overall median time from TAVI to PCI was 470 days (IQR 50–1285), and the majority of patients (80%) underwent PCI more than 30 days after TAVI. Radial access was used in 60% of procedures (62% in BEV vs. 59% in SEV, *p* = 0.808). The most commonly used guiding catheter was EBU (62%), with similar distribution between groups; the most frequent size and curve were EBU 6F (82%) and EBU 3.5 (84%), respectively.

Regarding target vessels, LAD PCI tended to be more frequent in the BEV group (59% vs. 41%, *p* = 0.129), whereas LCX PCI was significantly more frequent in the SEV group (54% vs. 29%, *p* = 0.035). The mean number of stents implanted was 1.4 ± 1 overall, with a non-significant trend toward a higher number in the BEV group (1.6 ± 1 vs. 1.1 ± 1, *p* = 0.075). Balloon angioplasty without stenting was performed in 16% of cases, and rotational atherectomy in 12%, without significant differences between groups. There was a trend toward more frequent use of guide extension catheters in the SEV group compared with the BEV group (23% vs. 9%, *p* = 0.092). In addition, procedure duration did not differ significantly between groups (60 ± 20 vs. 55 ± 18 min, *p* = 0.343).

### 3.3. PCI Procedural and Clinical Outcomes

**Figure 1:**
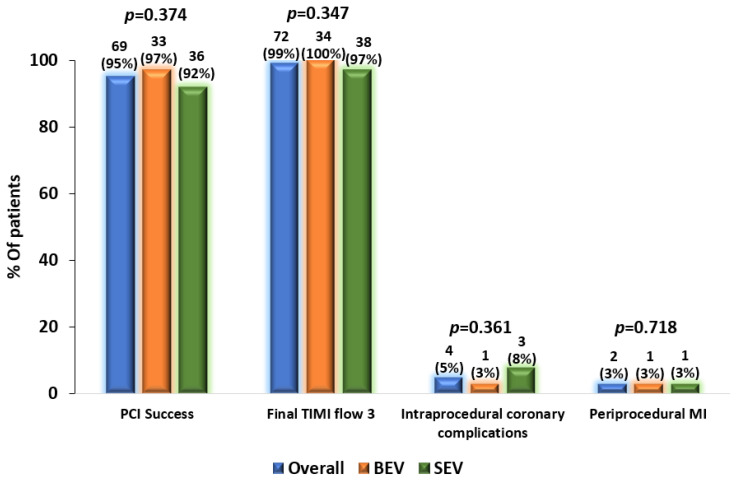
PCI Procedural outcomes.

**Figure 2:**
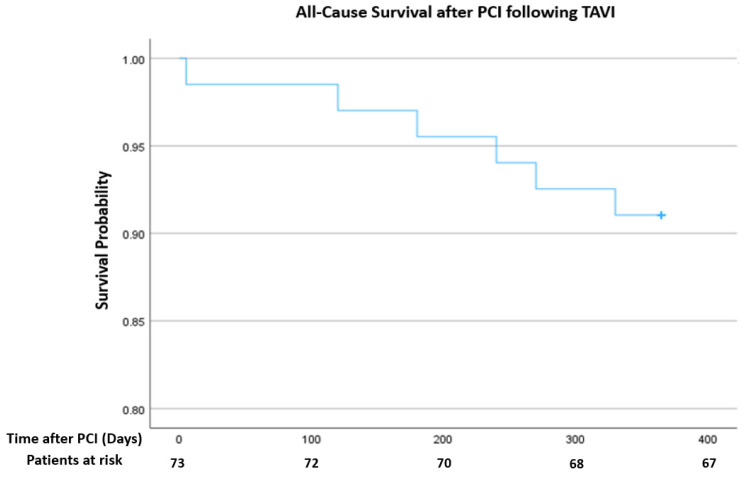
All-Cause Survival after PCI following TAVI.

**Figure 3:**
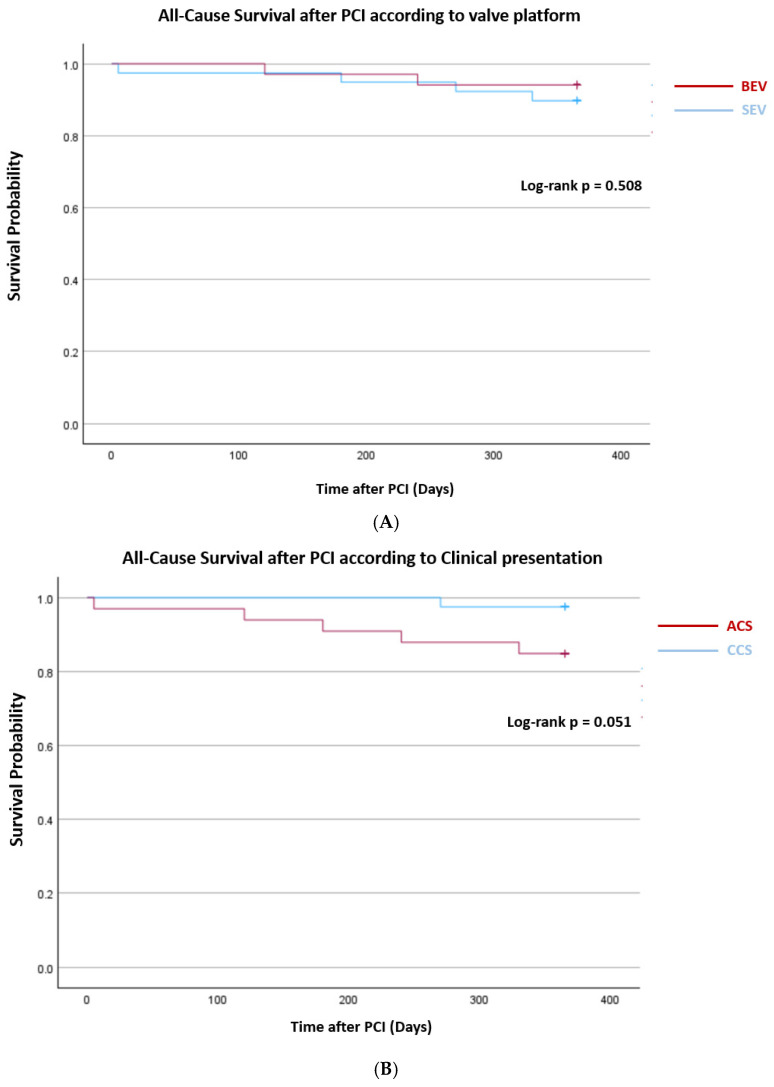
Kaplan–Meier estimates of 1-year all-cause survival after PCI following TAVI according to (**A**) valve platform and (**B**) clinical presentation.

PCI procedural and clinical outcomes are summarized in Table 4 and Figure 1. PCI success was high overall (95%), with rates of 97% in the BEV group and 92% in the SEV group (*p* = 0.374). Final TIMI 3 flow was achieved in 99% of cases (100% BEV vs. 97% SEV, *p* = 0.347). Intraprocedural coronary complications were infrequent (5% overall; 3% BEV vs. 8% SEV, *p* = 0.361) (Supplementary Table S1), and periprocedural myocardial infarction occurred in 3% of patients in both groups. Major bleeding occurred in 7% of cases without significant differences between platforms, including three cases of retroperitoneal haemorrhage and two cases of procedure-related anaemia requiring transfusion of 2–3 units of packed red blood cells. Only one in-hospital death occurred in the entire cohort (2%), in a patient from the SEV group due to cardiogenic shock and multiorgan failure following an anterior STEMI. At 1-year follow-up, overall mortality was 8% (Figure 2), with no statistically significant difference between the BEV and SEV groups (6% vs. 10%, *p* = 0.497). Kaplan–Meier analysis confirmed the absence of a significant difference in 1-year survival according to valve platform (log-rank *p* = 0.508; Figure 3A). Among the six patients treated with the Navitor platform, PCI success was achieved in five patients (83%). One intraprocedural coronary dissection occurred, final TIMI 3 flow was achieved in all patients, and no deaths were observed at 1-year follow-up.

In a subgroup analysis according to clinical presentation (CCS vs. ACS) (Table 5), there was no difference in valve platform distribution between groups (SEVs: 55% in CCS vs. 52% in ACS; *p* = 0.766). The most frequently treated vessel in CCS patients was the LAD (58%), whereas LCX PCI was more common in ACS presentations (42%). PCI success was high in both CCS and ACS groups (97% in CCS vs. 91% in ACS, *p* = 0.218), with final TIMI 3 flow achieved in 100% of CCS cases and 97% of ACS cases (*p* = 0.268). Intraprocedural coronary complications were infrequent (3% in CCS vs. 9% in ACS; *p* = 0.238). Finally, there was a trend toward higher 1-year mortality in patients presenting with ACS compared with CCS (15% vs. 3%, *p* = 0.061). Kaplan–Meier analysis similarly demonstrated a trend toward lower 1-year survival in ACS patients compared with CCS patients, although the difference did not reach statistical significance (log-rank *p* = 0.051; Figure 3B).

## 4. Discussion

In this single-center real-world cohort, PCI performed after TAVI demonstrated high procedural feasibility, with low rates of intraprocedural and clinical adverse events. Angiographic success was excellent, with nearly universal achievement of final TIMI 3 flow and a very low rate of intraprocedural coronary complications. Importantly, no statistically significant differences in procedural and clinical outcomes were observed between BEV and SEV platforms. In subgroup analysis according to clinical presentation, there was a numerical trend toward higher 1-year mortality among patients presenting with ACS compared with CCS (15% vs. 3%, *p* = 0.061). However, this exploratory finding should be interpreted cautiously given the limited sample size, lack of multivariable adjustment, and potential differences in baseline clinical characteristics between groups.

Concerns regarding coronary access after TAVI are often more pronounced with SEVs, given their taller frame and leaflet position, which may potentially interfere with selective coronary engagement. In contrast, BEV platforms usually have an intra-annular design and shorter frame height, which are generally considered to facilitate coronary re-access for PCI after TAVI.

According to the latest evidence reported in the CANNULATE TAVI EXPANDED study (*n* = 126), which evaluated coronary cannulation after Evolut FX SEV implantation using post-TAVI computed tomography (CT), coronary cannulation success was very high (100% for the left main coronary artery and 96% for the right coronary artery), and coronary misalignment emerged as a strong independent predictor of suboptimal coronary cannulation [16]. To date, no studies have directly compared PCI rates or procedural success between BEV and SEV platforms in patients undergoing PCI after TAVI. Available data suggest that although PCI is feasible with both valve types, coronary engagement following SEV implantation may require additional technical considerations, such as the need for guide extension catheters [14,17]. In one large analysis [18], procedural success of PCI was similar in patients with prior TAVI compared with propensity-matched patients without TAVI. However, patients with previous TAVI experienced higher rates of post-procedural stroke, bleeding and mortality. Tarantini et al. [19] demonstrated that selective coronary access after TAVI was significantly more feasible with short-frame BEVs (Sapien3 and Sapien 3 Ultra) compared with tall-frame SEVs, particularly Evolut Pro and Evolut Pro+. Costa et al. [20] evaluated coronary re-engagement after TAVI using SEVs. Unsuccessful coronary cannulation occurred in 5.5% of cases, predominantly among Evolut recipients, with severe commissural misalignment identified as a key predictor of impaired coronary access. Finally, in a large registry evaluating coronary access in ACS settings after TAVI [21], coronary angiography and PCI were feasible in the vast majority of patients. While selective coronary engagement was more challenging with long stent-frame prostheses, PCI success rates were high and did not differ according to valve type.

To the best of our knowledge, this is one of the first single-center studies directly comparing procedural characteristics and clinical outcomes of PCI performed after TAVI between BEV and SEV platforms. Despite concerns regarding coronary access with SEVs, we observed high procedural success, excellent angiographic results, and low complication rates across both valve types. While successful coronary cannulation is a prerequisite for PCI, it represents a distinct procedural endpoint. Our study specifically focused on PCI success and clinical outcomes rather than coronary cannulation itself. Importantly, no statistically significant differences in procedural or clinical outcomes were observed between platforms. However, the limited sample size precludes excluding clinically relevant differences between valve designs. Thus, our findings suggest that PCI after TAVI is feasible in both short- and long-stent transcatheter heart valve platforms when appropriate techniques are applied.

## 5. Limitations

This study presents several limitations. First, the relatively small sample size, particularly after stratification according to valve platform and the limited number of patients treated with specific devices such as Navitor, limits the statistical power of the study, reducing the ability to detect modest differences between groups. In addition, the inclusion of different valve generations within the BEV and SEV groups may have introduced device-related heterogeneity that could not be evaluated separately. Nevertheless, the study reflects real-world experience with PCI performed after TAVI. Second, for long stent platforms, commissural alignment was not systematically performed (as some TAVI procedures were performed particularly in earlier cases, before adoption of this technique), and no routine post-TAVI computed tomography was available to assess commissural alignment or valve implantation depth. In addition, detailed procedural data regarding coronary cannulation techniques, including selective versus non-selective engagement, catheter crossover, and other technical aspects of coronary access, were not systematically collected. Furthermore, the long study period (2016–2024) encompassed changes in valve technology, operator experience, and procedural techniques, which may have introduced temporal confounding. Third, we included only patients undergoing PCI after TAVI; therefore, the strategy of treating the coronaries and aortic valve together was not analysed, and no comparison between PCI performed before versus after TAVI is provided. Fourth, given the observational and non-randomized design, differences in baseline characteristics, including target vessel distribution, as well as potential unmeasured confounders, may have influenced procedural and clinical outcomes. Fifth, only patients who underwent PCI after TAVI were included. Therefore, unsuccessful coronary angiography or PCI attempts, as well as procedures deferred because of inability to obtain coronary access, were not captured, and the reported feasibility should be interpreted within the context of this selected study population. Furthermore, because of the limited sample size and low number of clinical events, adjusted multivariable or propensity-score analyses were not considered statistically appropriate. In addition, follow-up clinical outcomes such as myocardial infarction, repeat revascularization, heart failure hospitalization, and major adverse cardiovascular events were not systematically collected and therefore could not be analyzed. Finally, as a single-center experience, the findings may not be generalizable to institutions with different patient populations or procedural strategies.

## 6. Conclusions

In conclusion, our findings suggest that PCI after TAVI is feasible in selected patients with BEV and SEV platforms across different clinical scenarios. Although coronary cannulation has been reported in previous studies, the success of PCI is rarely mentioned, and it can sometimes be more challenging when selective cannulation is difficult to achieve. Our research aimed to identify the challenges of PCI after TAVI in stable and acute scenarios, highlighting the high rate of successful PCI despite the need for technical compromise. However, these findings should be interpreted in the context of the observational single-center design and limited sample size and warrant confirmation in larger multicenter studies.

**Clinical Perspectives:**

- PCI after TAVI demonstrated high feasibility, with a procedural success rate of 95% and final TIMI 3 flow in 99% of cases.
- Procedural success and clinical outcomes were comparable between balloon-expandable and self-expandable valve platforms, despite potential concerns regarding coronary access with supra-annular valves.
- Clinical presentation influenced outcomes, with a trend toward higher 1-year mortality in patients presenting with ACS compared with CCS.

## Figures and Tables

**Table 1:** Baseline characteristics.

Characteristic	Overall	BEV	SEV	*p*-Value
*n*	73	34	39	
Age, years, (mean ± SD)	81 ± 6	82 ± 5	81 ± 7	0.615
Gender, male *n* (%)	47 (64)	26 (76)	21 (54)	0.044
**Cardiovascular Comorbidities**				
Dyslipidemia, *n* (%)	35 (48)	15 (44)	20 (51)	0.541
Hypertension, *n* (%)	55 (75)	26 (76)	29 (74)	0.835
Diabetes mellitus, *n* (%)	18 (25)	7 (21)	11 (28)	0.451
BMI, (mean ± SD)	26.2 ± 3.7	26.4 ± 4.1	26.1 ± 3.5	0.673
Coronary artery disease, *n* (%)	53 (72)	23 (68)	30 (77)	0.375
Prior PCI, *n* (%)	30 (41)	11 (32)	19 (49)	0.156
Prior CABG, *n* (%)	15 (21)	5 (15)	10 (26)	0.249
Prior CVA/TIA, *n* (%)	3 (4)	1 (3)	2 (5)	0.552
PVD, *n* (%)	11 (15)	5 (15)	6 (15)	0.936
Atrial fibrillation, *n* (%)	14 (19)	8 (23)	6 (15)	0.378
**Surgical Risk**				
Euroscore II, median (Q1, Q3)	5.2 (3.1, 8.1)	5.0 (3.0, 7.4)	5.2 (3.2, 8.8)	0.167
STS Score, median (Q1, Q3)	4.4 (2.9, 6.7)	3.8 (2.4, 6.4)	4.8 (3.6, 6.9)	0.151

BEV = Balloon-expandable valve; SEV = Self-expandable valve; BMI = Body Mass Index; PCI = Percutaneous coronary intervention; CABG = Coronary artery bypass graft; CVA = Cerebrovascular Accident; TIA = Transient ischemic attack; PVD = Peripheral vascular disease.

**Table 2:** TAVI procedural characteristics.

Characteristic	Overall	BEV	SEV	*p* Value
*n*	73	34	39	
**Valve size, mm**				
23	9 (12)	5 (15)	4 (10)	0.564
25	3 (4)	0 (0)	3 (8)	0.147
26	28 (38)	20 (59)	8 (21)	<0.001
27	2 (3)	0 (0)	2 (5)	0.282
29	27 (37)	9 (26)	18 (46)	0.082
34	3 (4)	0 (0)	3 (8)	0.147
35	1 (1)	0 (0)	1 (3)	0.534
Transfemoral access, *n* (%)	71 (97)	32 (94)	39 (100)	0.125
Device success *, *n* (%)	67 (92)	31 (91)	36 (92)	0.861
Concomitant PCI during TAVI, *n* (%)	4 (5)	1 (3)	3 (8)	0.361
Post-TAVI mean gradient, (mean ± SD)	10 ± 4	12 ± 4	8 ± 4	<0.001
Post-TAVI EF, %, (mean ± SD)	56 ± 12	57 ± 13	55 ± 12	0.501
Peri-procedural stroke, *n* (%)	2 (3)	1 (3)	1 (3)	0.718
Permanent Pacemaker, *n* (%)	7 (10)	3 (9)	4 (10)	0.556

BEV = Balloon-expandable valve; SEV = Self-expandable valve; TAVI = Transcatheter Aortic Valve Implantation; EF = Ejection fraction; PVL = Paravalvular leak. * Defined as absence of procedural mortality, correct position of a single prosthetic valve with a mean aortic gradient < 20 mmHg or peak velocity < 3 m/s and no moderate or severe prosthetic aortic valve regurgitation.

**Table 3:** PCI procedural characteristics.

Characteristic	Overall	BEV	SEV	*p* Value
*n*	73	34	39	
**Indication for PCI**				
CCS, *n* (%)	40 (55)	18 (53)	22 (56)	0.766
ACS, *n* (%)	33 (45)	16 (47)	17 (44)	0.766
**TAVI to PCI, days, median (Q1, Q3)**	470 (50, 1285)	400 (53, 1282)	545 (48, 1246)	0.355
TAVI to PCI > 30 Days, *n* (%)	58 (80)	29 (85)	29 (74)	0.249
Vascular approach, radial *n* (%)	44 (60)	21 (62)	23 (59)	0.808
**Guiding Catheter**				
EBU, *n* (%)	45 (62)	21 (62)	24 (62)	0.984
EBU Size 6F	37 (82)	16 (76)	21 (87)	0.44
EBU Curve 3.5	38 (84)	19 (90)	19 (79)	0.42
JL/JR, *n* (%)	16 (22)	7 (21)	9 (23)	0.798
Other *, *n* (%)	13 (18)	6 (17)	7 (18)	0.973
Guide extension catheter, *n* (%)	12 (16)	3 (9)	9 (23)	0.092
**Target vessel**				
LM PCI, *n* (%)	17 (23)	8 (24)	9 (23)	0.964
LAD PCI, *n* (%)	36 (49)	20 (59)	16 (41)	0.129
LCX PCI, *n* (%)	31 (42)	10 (29)	21 (54)	0.035
RCA PCI, *n* (%)	12 (16)	8 (24)	4 (10)	0.127
Vein or arterial graft PCI, *n* (%)	3 (4)	1 (3)	2 (5)	0.552
Number of stents, (mean ± SD)	1.4 ± 1	1.6 ± 1	1.1 ± 1	0.075
Balloon angioplasty only, *n* (%)	12 (16)	4 (12)	8 (21)	0.314
Rotablation, *n* (%)	9 (12)	6 (18)	3 (8)	0.197
Contrast volume, ml, (mean ± SD)	166 ± 62	164 ± 61	167 ± 64	0.419
Procedure duration, min, (mean ± SD)	58 ± 19	55 ± 18	60 ± 20	0.343

BEV = Balloon-expandable valve; SEV = Self-expandable valve; TAVI = Transcatheter Aortic Valve Implantation; PCI = Percutaneous Coronary Intervention; CCS = Chronic Coronary Syndrome; ACS = Acute Coronary Syndrome; LM = Left Main coronary artery; LAD = Left Anterior Descending artery; LCX = Left Circumflex artery; RCA = Right Coronary artery. * Other: Included Amplatz Left, Amplatz Right, multipurpose, and left internal mammary artery (LIMA) catheters.

**Table 4:** PCI Procedural and clinical outcomes.

Characteristic	Overall	BEV	SEV	*p* Value
*n*	73	34	39	
PCI success *, *n* (%)	69 (95)	33 (97)	36 (92)	0.374
Final TIMI flow 3, *n* (%)	72 (99)	34 (100)	38 (97)	0.347
Intraprocedural coronary complications †, *n* (%)	4 (5)	1 (3)	3 (8)	0.361
Periprocedural myocardial infarction, *n* (%)	2 (3)	1 (3)	1 (3)	0.718
Major bleeding ‡, *n* (%)	5 (7)	3 (9)	2 (5)	0.434
Post PCI EF, %, (mean ± SD)	54 ± 12	54 ± 12	53 ± 12	0.929
Post PCI Mean gradient (aortic valve), (mean ± SD)	11 ± 7	14 ± 6	9 ± 7	0.005
In-hospital death, *n* (%)	1 (1)	0 (0)	1 (3)	0.534
1-year death, *n* (%)	6 (8)	2 (6)	4 (10)	0.497

BEV = Balloon-expandable valve; SEV = Self-expandable valve. * Procedural Percutaneous Coronary Intervention (PCI) success after TAVI was defined as successful stent or drug-coated balloon implantation with final TIMI 3 flow and no intraprocedural complications. † Intraprocedural coronary complications were defined as a composite of in-stent thrombosis, slow/no-reflow, distal embolization, coronary dissection, coronary perforation, or intra-procedural death. ‡ Major bleeding was defined according to the Valve Academic Research Consortium-2 (VARC-2) criteria.

**Table 5:** Procedural and clinical outcomes according to clinical presentation (CCS vs. ACS).

Characteristic	CCS	ACS	*p* Value
*n*	40	33	
SEV, *n* (%)	22 (55)	17 (52)	0.766
TAVI to PCI > 30 Days, *n* (%)	29 (73)	29 (88)	0.106
Vascular approach, radial *n* (%)	25 (62)	19 (58)	0.669
**Guiding Catheter**			
EBU, *n* (%)	23 (58)	22 (67)	0.423
JL/JR, *n* (%)	12 (30)	4 (12)	0.066
Other, *n* (%)	6 (15)	7 (21)	0.490
**Target vessel**			
LM PCI, *n* (%)	11 (27)	6 (18)	0.349
LAD PCI, *n* (%)	23 (58)	13 (39)	0.124
LCX PCI, *n* (%)	17 (43)	14 (42)	0.995
RCA PCI, *n* (%)	7 (17)	5 (15)	0.788
Vein or arterial graft PCI, *n* (%)	1 (3)	2 (6)	0.427
Number of stents, (mean ± SD)	1.5 ± 1.2	1.3 ± 1.0	0.324
Balloon angioplasty only, *n* (%)	8 (20)	4 (12)	0.366
Rotablation, *n* (%)	4 (10)	5 (15)	0.505
Contrast volume, ml, (mean ± SD)	173 ± 74	158 ± 46	0.287
Procedure duration, min, (mean ± SD)	59 ± 20	57 ± 19	0.719
PCI success *, *n* (%)	39 (97)	30 (91)	0.218
Final TIMI flow 3, *n* (%)	40 (100)	32 (97)	0.268
Intraprocedural coronary complications †, *n* (%)	1 (3)	3 (9)	0.238
Major bleeding ‡, *n* (%)	3 (8)	2 (6)	0.592
In-hospital death, *n* (%)	0 (0)	1 (3)	0.452
1-year death, *n* (%)	1 (3)	5 (15)	0.061

SEV = Self-expandable valve; TAVI = Transcatheter Aortic Valve Implantation; PCI = Percutaneous Coronary Intervention; CCS = Chronic Coronary Syndrome; ACS = Acute Coronary Syndrome; LM = Left Main coronary artery; LAD = Left Anterior Descending artery; LCX = Left Circumflex artery; RCA = Right Coronary artery. * Procedural Percutaneous Coronary Intervention (PCI) success after TAVI was defined as successful stent or drug-coated balloon implantation with final TIMI 3 flow and no intraprocedural complications. † Intraprocedural coronary complications were defined as a composite of in-stent thrombosis, slow/no-reflow, distal embolization, coronary dissection, coronary perforation, or intra-procedural death. ‡ Major bleeding was defined according to the Valve Academic Research Consortium-2 (VARC-2) criteria.

## Data Availability

The datasets used and/or analysed during the current study are available from the corresponding author on reasonable request.

## Supplementary Materials

The following supporting information can be downloaded at https://www.mdpi.com/article/10.3390/jcm15145616/s1, Table S1. Intraprocedural coronary complications.

## Abbreviations

TAVI	Transcatheter Aortic Valve Implantation
BEV	Balloon-expandable valve
SEV	Self-expandable valve
CCS	Chronic coronary syndrome
ACS	Acute coronary syndrome
