# Supplementary material for: Feasibility and Outcomes of Percutaneous Coronary Intervention After TAVI: A Comparison Between BEV and SEV Platforms
Source: J Clin Med. 2026 Jul 17;15(14):5616. doi: 10.3390/jcm15145616 (PMC13413311; doi:10.3390/jcm15145616)
Supplement: Supplementary file 1 [file jcm-15-05616-s001.zip › jcm-4415506-supplementary.pdf]

Supplementary material

Table S1 – Intraprocedural coronary complications

Characteristic	Overall	BEV	SEV	P value
n	73	34	39	
Vessel dissection, n (%)	2 (3)	0 (0)	2 (5)	0.282
Vessel perforation, n (%)	0 (0)	0 (0)	0 (0)	-
Distal embolization, n (%)	1 (1)	0 (0)	1 (3)	0.534
Slow Flow, n (%)	1 (1)	0 (0)	1 (3)	0.534
In stent thrombosis, n (%)	1 (1)	1 (3)	0 (0)	0.466
Intra-procedural death, n (%)	0 (0)	0 (0)	0 (0)	-

BEV=Balloon-expandable valve; SEV=Self-expandable valve;
